# Role of Somatic Testicular Cells during Mouse Spermatogenesis in Three-Dimensional Collagen Gel Culture System

**Published:** 2014-02-03

**Authors:** Noushafarin Khajavi, Mohammad Akbari, Farid Abolhassani, Ahmad Reza Dehpour, Morteza Koruji, Mehryar Habibi Roudkenar

**Affiliations:** 1 Department of Anatomical Sciences, School of Medicine, Tehran University of Medical Sciences, Tehran, Iran; 2 Department of Pharmacology, School of Medicine, Tehran University of Medical Sciences, Tehran, Iran; 3 Department of Anatomical Sciences, School of Medicine, Iran University of Medical Sciences, Tehran, Iran; 4 Research Center, Iranian Blood Transfusion Organization, Tehran, Iran

**Keywords:** Mouse, Spermatogonia, Spermatogenesis, Cell Culture Technique

## Abstract

**Objective:**

Spermatogonial stem cells (SSCs) are the only cell type that can restore fertility to an infertile recipient following transplantation. Much effort has been made to develop a protocol for differentiating isolated SSCs *in vitro*. Recently, three-dimensional (3D) culture system has been introduced as an appropriate microenvironment for clonal expansion and differentiation of SSCs. This system provides structural support and multiple options for several manipulation such as addition of different cells. Somatic cells have a critical role in stimulating spermatogenesis. They provide complex cell to cell interaction, transport proteins and produce enzymes and regulatory factors. This study aimed to optimize the culture condition by adding somatic testicular cells to the collagen gel culture system in order to induce spermatogenesis progression.

**Materials and Methods:**

In this experimental study, the disassociation of SSCs was performed by using a two-step enzymatic digestion of type I collagenase, hyaluronidase and DNase. Somatic testicular cells including Sertoli cells and peritubular cells were obtained after the second digestion. SSCs were isolated by Magnetic Activated Cell Sorting (MACS) using GDNF family receptor alpha-1 (Gfrα-1) antibody. Two experimental designs were investigated. 1. Gfrα-1 positive SSCs were cultured in a collagen solution. 2. Somatic testicular cells were added to the Gfrα-1 positive SSCs in a collagen solution. Spermatogenesis progression was determined after three weeks by staining of synaptonemal complex protein 3 (SCP3)-positive cells. Semi-quantitative Reverse Transcription PCR was undertaken for SCP3 as a meiotic marker and, Crem and Thyroid transcription factor-1 (TTF1) as post meiotic markers. For statistical analysis student t test was performed.

**Results:**

Testicular supporter cells increased the expression of meiotic and post meiotic markers and had a positive effect on extensive colony formation.

**Conclusion:**

Collagen gel culture system supported by somatic testicular cells provides a microenvironment that mimics seminiferous epithelium and induces spermatogenesis *in vitro*.

## Introduction

The increasing number of infertile patients has provoked a number of studies to investigate the cellular mechanisms during spermatogenesis and to generate male gametes *in vitro* (1, 2). Although *in vitro* spermatogenesis is considered as an important topic in reproductive biology and its regulatory mechanism has been detected in different approaches, entry into meiosis and the condition in which meiosis occurs during spermatogenesis is still poorly understood. Therefore, indicating an optimal culture condition for meiotic and post meiotic spermatogonial stem cell (SSC) differentiation is extremely difficult (3). Recently, miscellaneous evidences have demonstrated that SSCs survive and enter into meiosis under improved culture condition (4, 5).

Utilizing several culture conditions has led to the understanding that the spatial arrangement of the testicular cells is extremely important during SSC differentiation (6). Conventional cell culture or two dimensional culture systems (2D), has provided a thin layer with gelatin, collagen or other matrix substances. This culture system does not provide the spatial arrangement present in the natural environment. Meiotic cells in the natural environment are engulfed in sertoli cells as large interconnected clones with no contact to the basement membrane and such a sophisticated structure cannot be provided by 2D culture system. Other researchers have shown that three-dimensional culture (3D) as an improved culture system can provide a great opportunity for spermatogonial stem cell-somatic testicular cell contact which is immensely important during spermatogenesis stages. Soft agar culture system (SACS), collagen gel matrix and Methylcellulose culture system (MCS), by providing a thick layer for embedding SSCs and somatic testicular cells, produce a microenvironment which might resemble the seminiferous epithelium and avoid the ischemia in a long-term testicular tissue culture (4, 5, 7). Recently, new studies have demonstrated the importance of somatic cells in stimulating SSCs progression and survival during culture. A 3D culture system supported with somatic cells could provide an improved culture system by creating physical and paracrine support for allowing SSCs to enter meiosis (1).

Although the critical role of somatic testicular cells in spermatogenesis induction has been demonstrated in several reports, the involvement of these cells in meiotic progression during 3D culture system of collagen gel matrix remains unclear.

Taking everything into consideration, *in vitro* conditions for complete spermatogenesis is very far from routine methodology. So far, there is no study to address the effect of somatic testicular cells in meiotic promotion under a collagen gel matrix. This study was aimed to examine the effect of co-culturing these cells on SSC meiotic differentiation in a collagen gel culture system.

## Materials and Methods

### Animals

Testes were obtained from 7 day-old postpartum Balb-c mice from Pasteur Institute. At this age, meiotic germ cells are not detectable in the testis and seminiferous epithelium contains proliferating sertoli cells and spermatogonia. Animal experimental procedures were performed according to the ethical guidelines on handling experimental animals.

### Testicular cell isolation

After removing testis from the scrotum, decapsulated tissue was minced mechanically by multiple aspirations through pipette tips and after complete disassociation of the tubules they were transferred into the culture medium Dulbecco’s Modified Eagle Medium (DMEM/HAMF12; Gibco, USA). Digestion was conducted in two steps. In the first step, in order to obtain testicular cell fraction only collagenase Type 1A (1 mg/ml, Sigma, Germany) was added to the medium. Digestion was performed for 10 minutes in a shaking water bath operated at 110 cycles per minute. The fraction was separated by sedimentation at unit gravity. Tubules were allowed to settle by gravity and washed by phosphate buffered saline (PBS). Supernatant containing the interstitial cells was removed and the cell fraction was stored in DMEM/ HAM F12. In the next step, to obtain a fraction consisting of large proportion of germ cells, sertoli cells and peritubular cells, the fragments obtained after the first digestion were washed in DMEM and digested in a mixture of collagenase type IA (1 mg/ mL, Sigma, Germany), DNase (0.5mg/ml, Sigma, Germany), and hyaluronidase (0.5 mg/ml, Sigma, Germany). Digestion was performed at 32˚C for about 10 minutes. After washing, the single-cell suspension mainly consisted of germ cells, sertoli cells and also Peritubular cells. The efficiency of the digestion was evaluated microscopically.

### Magnetic labeling and separation of cells

Aliquots of single-cell suspensions with a concentration of around 7.5×107 cells/mL were used for indirect labeling. In the first step of the procedure, the cells were incubated with a polyclonal rabbit anti– Gfrɑ-1 immunoglobulin G (IgG) antibody (H-70, diluted 1:20; Santa Cruz Biotechnology, Santa Cruz, California) for 15 minutes at 4˚C. After washing the cells with PBS containing 2 mM EDTA (Sigma, Germany) and 0.5% fetal calf serum (Gibco, USA), the cells were incubated with anti-rabbit IgG biotin conjugate (B-8895; Sigma) for 15 minutes at 4˚C. The cell suspension was washed again and labeled with anti-biotin MicroBeads (dilution 1:5; Miltenyi, Canada) for 15 minutes and washed. Medium size (MS) separation column (Miltenyi) was used for SSC isolation. At first, the column was placed in a strong magnetic field and then flushed with degassed buffer. After resuspending the Gfrɑ-1–labeled cell in degassed buffer, it was poured into the column. While unlabeled cells passed through the magnetic field, Gfrɑ-1–positive cells were retained. The column was washed 3 times with degassed buffer in order to remove unlabeled cells from the column. After removing the column from the magnet, it was washed with 500 μL degassed buffer in order to flush the cells out of the column. Trypan blue staining was used to evaluate the MAC-sorted cells viability.

### Flow cytometry

Sorted cell suspension was stained with FITCconjugated anti-rabbit antibody (F-0382; Sigma, Germany) for 30 minutes at 4˚C. FITC-positive cells were analyzed on a Beckman Coulter flow cytometer FC500 (Krefeld, Germany) equipped with a 15-mW argon-ion laser at an excitation wavelength of 488 nm.

### Immunofluorescence staining

In order to analyze Gfrɑ-1 expression in unsorted and enriched MAC-sorted fractions, cell suspensions were stained for Gfrɑ-1 with an FITC-labeled secondary antibody (Sigma) in combination with Hoechst 33528 for 30 minutes. The results were documented by digital imaging using a fluorescence microscope

For meiotic cell progression analysis, the entire well with all colonies and cells were transferred to a cassette and fixed in 4% paraformaldehyde for 24 hours at 6-8˚C. After fixation, collagen was washed in 30% (24 hours) and 50% (24 hours) ethanol and embedded in paraffin by using an automated processor. Cultured cells were cut into sections of 5-7 μm and deparaffinized. Before the primary antibody was applied, nonspecific background staining was blocked. PBS containing 0.05% casein and the relevant antibody for the relevant IgG isotope was utilized. Thereafter, colonies were incubated with primary antibody rabbit anti synaptonemal complex protein 3 (SCP3) (diluted 1:500 in PBS; Abcam, 15093), overnight at 4˚C in a moist chamber. The slides were rinsed in PBS and incubated with secondary antibody biotinylated goat anti-rabbit (diluted 1:150 in PBS; Zymed) for 2 hours at room temperature. The slides were rinsed again with PBS and were incubated with fluorescein isothiocyanate (FITC)-conjugated streptavidin (diluted 1:50 in PBS; Southern Biotech, Birmingham, AL, USA) for 3 hours in the dark. After rinsing in PBS and letting to dry, DAPI staining was performed and cells were observed by digital imaging using a fluorescence microscope. Negative controls were also analysed for each specimen utilizing PBS/casein/relevant IgG isotype instead of primary antibodies. Five different microscopic fields were chosen, and the number of SCP3 positive cells was counted. Counting was carried out blindly by three observers.

### Three-dimensional culture system (collagen gel matrix)

In order to obtain collagen, in the first step tails of rats (44-48 days old) were cut and skinned. Then, individual tendon fibers were removed through the surrounding fascia out of the tail. Collagen tendons sterilization was performed by 70% ethanol. After Collagen tendons sterilization, they were placed in 0.01% acetic acid for complete dissolution. The mixture was centrifuged at 15000 g for 30 minutes and supernatant was then used as collagen solution. 24 well plate has been utilized for this study. Collagen solution was mixed with DMEM/F12 containing fetal bovine serum and culture cells. In order to avoid heat-induced cellular stress and premature collagen coagulation, collagen and cells were mixed at 37˚C. The standard cell culture incubator with 37˚C and 5% CO2 was utilized for this study.

### Experimental protocol

In the present study two experimental designs were investigated. In the first group, Gfrɑ-1 positive SSCs obtained by magnetic cell sorting were cultured in collagen solution without somatic testicular cells support. In the second group, somatic testicular cells including Sertoli and peritubular cells, which had been obtained after the second digestion, were added to the Gfrɑ-1 positive SSCs in a collagen solution.

### Total RNA extraction, reverse transcription polymerase chain reaction and cDNA synthesis

After the second digestion and after the MACS isolation, total RNA was extracted from single cell suspensions using the TriPure Isolation Reagent (Roche, Germany) according to the manufacturer’s instructions. RNA extraction was also performed for the cultured cell after 7 and 21 days. Cell-collagen gel mixture was dissolved by using type 1 collagenase (Sigma) to release embedded cells from the collagen gel matrix. The mixture was rinsed with PBS and total RNA was extracted from the cells using the TriPure Isolation Reagent (Roche, Germany) according to the manufacturer’s instructions. The quantity of the extracted RNA was determined with a ND-1000 Spectrophotometer (NanoDrop Technologies, Wilmington, USA). cDNA synthesis kit (BIONEER, Korea) was used to synthesize cDNA. The reverse transcription of RNA was performed in a total volume of 20 μl for 12 cycles of three steps (25˚C for 30 seconds, 45˚C for 4 minutes, and 55˚C for 30 seconds). Analyzing gene expression levels was performed by polymerase chain reaction using the forward and reverse primers listed in table 1. PCR amplifications were conducted under the following conditions: 3 minutes at 95˚C, 33 cycles of 95˚C, 30 seconds; annealing temperature has been mentioned in table 1 for HOW LONG sec; and 72˚C, 45 seconds) and 72˚C for 7 minutes for final extension.

Then, the expression pattern of the genes was analyzed by UVIdoc Gel Documentation System (Cambridge, UK). Signal intensities of reverse transcription products were measured by SCION IMAGE analysis software.

### Statistical analysis

For statistical analysis of data among all the groups, student t test was performed. P value ≤0.05 considered statistically significant.

**Table 1:** Primer sequences, sizes and annealing temperatures for mRNA expression of markers of different mouse spermatogenic stages and somatic testicular cells used .


Stage	Target	Primer	Product size	Annealing temp.

**Pre-meiotic**	GFRα-1	Forward: GGCCTACTCGGGACTGATTGG Reverse: GGGAGGAGCAGCCATTGATTT	462bp	57
**Pre-meiotic**	OCT-4	Forward: AGAAGGAGCTAGAACAGTTTGC Reverse: CGGTTACAGAACCATACTCG	416bp	57
**Meiotic**	SCP3	Forward: ACAACAAGAGGAAATACAGAA Reverse: GAGAGAACAACTATTAAAACA	618bp	48
**Post-meiotic**	Crem	Forward: CTAGCACGGGGCAATACAAT Reverse: TCTGCTAGTTGCTGGGGACT	358bp	50
**Post-meiotic**	TTF1	Forward: GGCTTGTTCCACTGAGAAGC Reverse: TACATGCGTCTGATGGTGGT	225bp	52
**Sertoli cells**	ABP	Forward: GGAGAAGAGAGACTCTGTGG Reverse: GCTCAAGACCACTTTGACTC	900bp	57
**Peritubular cells**	α-Sm	Forward: CATCAGGCAGTTCGTAGCTC Reverse: CGATAGAACACGGCATCATC	524bp	57
**Housekeeping**	β-actin	Forward: TCATGAAGATCCTCACCGAG Reverse: TTGCCAATGGTGATGACCTG	190bp	50


## Results

### Evaluation of isolated tubular cells

**Fig 1:**
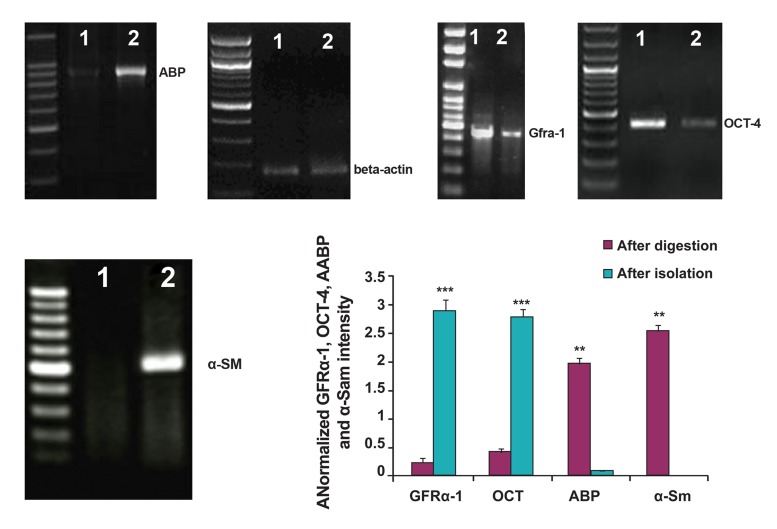
Expression analysis of premeiotic and somatic testicular marker genes GFRα-1, OCT4, ABP and α-Sm.1, after MACS isolation 2, after second digestion. β-actin was used as an internal control. Densitometric analysis is shown in the histogram.

After the second digestion, single cells were examined by RT-PCR for characterization of premeiotic cells, Sertoli cells and Peritubular cells. Our results revealed the expression of GFRα-1 and OCT-4 as premeiotic markers, Androgen Binding Protein (ABP) as a specific marker of Sertoli cells and α-Sm as a specific marker of peritubular cells. After MACS isolation, the cell suspension was investigated again for the expression of premeiotic, Sertoli and Peritubular cells markers. We observed a marked increase in the expression of premeiotic markers in isolated cells, while the expression of somatic cell markers were not statistically significant (Table 2, Fig 1).

**Table 2:** Expression of mouse spermatogenic and somatic testicular cells markers after digestion, after isolation and after 3D culture.


		Pre-meiotic		Somatic cells		Meiotic	Post-meiotic	

		GFRα-1	OCT-4	ABP	α-Sm	SCP3	Crem	TTF-1
**After digestion**		0.245±0.09	0.434±0.07	1.98±0.1	2.531±0.12	0.00	0.00	0.00
**After MACS isolation**		2.881±0.2	2.783±0.14	0.095±0.01	0.00	0.00	0.00	0.00
**P**		***	***	**	**	n.d.	n.d.	n.d.
**After culture**	**Control**	-	-	-	-	0.788±0.12	1.233±0.1	1.588±0.15
	**Co-culture**	-	-	-	-	1.751±0.2	2.144±0.13	2.666±0.15
**P**						*	*	*


n.d; Not determined, *; P<0.05, **; P<0.01 and ***; P<0.001.

### Flow cytometric and immunocytochemical evaluation of Spermatogonial Stem Cell marker

**Fig 2:**
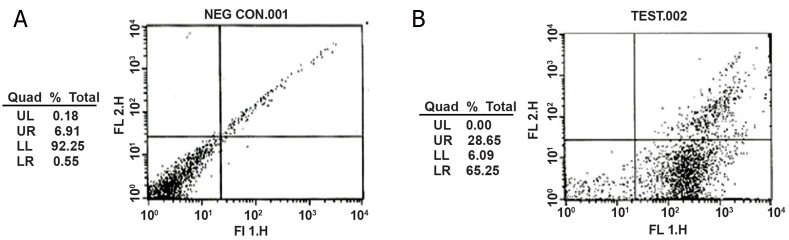
Flow cytometric analysis of Gfrɑ-1 positive SSCs. Quadrant 4 represents Gfrɑ-1 positive cells. A. Control and b. Sorted cells.

Flow cytometric analysis was used to investigate the markers to be efficient for undifferentiated spermatogonial stem cell isolation after MACS with anti-Gfrα-1 (Fig 2). Flow cytometric analysis indicated that a high percentage of undifferentiated SSCs (up to 65 %) could be isolated by Gfrα-1 marker.

**Fig 3:**
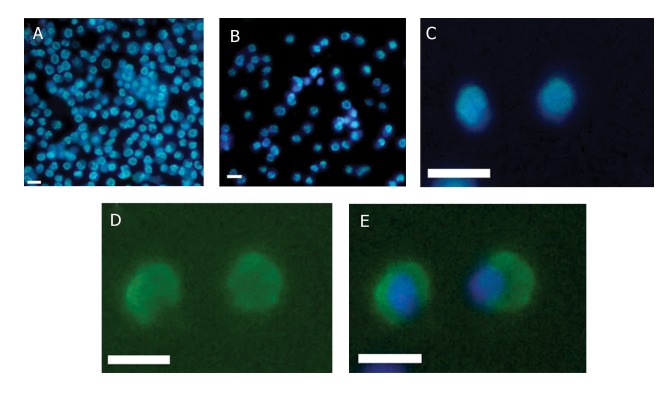
Images of fluorescence-labeled cells, DNA staining by Hoechst (blue) Gfrɑ-1–positive cells [FITC] (green). A. A heterogeneous cell suspension observed in unsorted cell fraction before the separation procedure. B. A homogeneous cell suspension is observed in the sorted fraction. C. The mentioned fractions are shown after immunofluorescent labeling with anti–Gfrɑ-1 (staining by Hoechst). D. labeling with anti-Gfrɑ-1 and E. merge.

Gfrɑ-1 expression also has been analyzed in unsorted and sorted fractions. Cell suspensions were stained for Gfrα-1 with an FITC-labeled secondary antibody (Sigma, Germany) with Hoechst 33528. The unsorted fraction contained a heterogeneous suspension of living cells (Fig 3A). After separation with MACS, sorted fraction contained cells with similar sizes and shapes, comparable nuclear-cytoplasm ratio and nuclear morphology (Fig 3B). Microscopic imaging of immunocytochemical staining demonstrated presence of a high number of Gfrɑ-1–positive cells in the sorted fraction in comparison with unsorted suspensions (Fig 3C, D, E)

### Spermatogonial stem cells development evaluation with and without supporter cells

**Fig 4:**
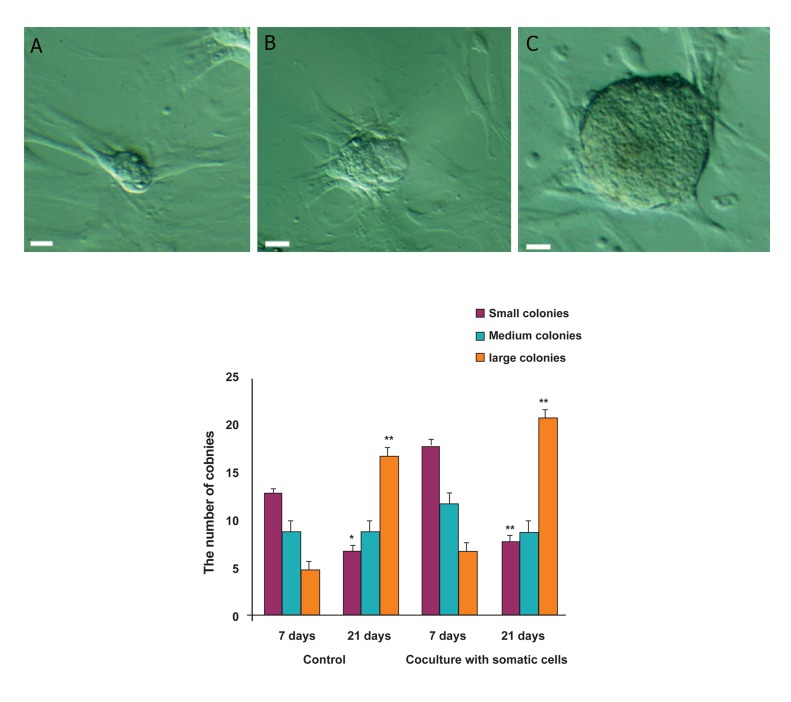
Images of (A) Small, (B) Medium and (C) Large colonies, (D) .The capacity of supporter cells to form Small, Medium or Large colonies was examined after 7 and 21 days of culture (*; p<0.05 and **; p<0.01).

Structure of colonies was investigated after 7 and 21 days of 3D culture. Colonies of various sizes were detected and encountered (Fig 4). Size of the colonies were measured by Image J software and colonies were designated as small when they were smaller than 100 μm2 and as medium when the size was between 100 to 500 μm2 and large when they were larger than 500 μm2. We showed that after 7 days of culture, the number of small colonies was remarkably more than medium colonies. There were only few numbers of large colonies in the culture system with or without supporter cells. After 21 days of both culture conditions, the number of large colonies showed a great increase (p<0.01) while a significant decline was detected in the number of small colonies (p<0.05). Comparing culture conditions revealed that the number of colonies in the absence of supporter cells was remarkably less than colonies in the presence of supporter cells (p<0.05).

**Fig 5:**
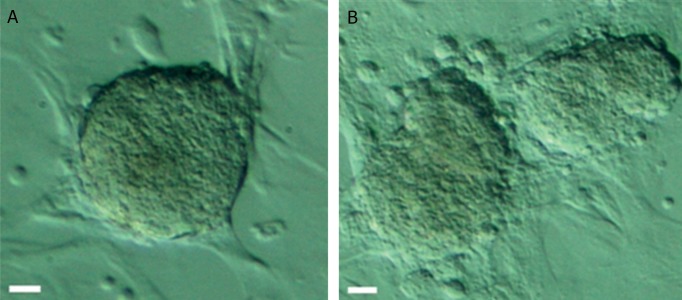
Images of clonal expansion of 3D-cultured after different culture periods: A. without supporter cells and B. with supporter cells.

Colony morphological analysis in the gel phase showed a different pattern over times in response to the two different conditions. Without supporter cells, colonies in the gel phase had round shape with sharp edges and they were heavily compacted (Fig 5A). With supporter cells, colonies in the gel phase were less dense and cells in the edges were in loose contact with the colony (Fig 5B).

To analyze spermatogenic development during culture in these two experimental designs, mRNA was isolated and semi-quantitative RT-PCR was performed. SCP3 as a meiotic and, Crem and TTF1 as post meiotic markers along with beta-actin as a housekeeping gene were examined in this study. Several evidences have demonstrated that the expression of SCP3 increases during meiotic division. SCP3 is required for synaptonemal complex assembly, chromosome synapsis and male fertility (8). Crem is considered also as a post meiotic marker at the stage of round spermatids. Immunohistochemical studies have shown that Crem is present in the spermatogenic stage of round spermatids (1, 4). As an additional marker to determine meiotic processes *in vitro*, TTF1 has been introduced. TTF1 binds to DNA as a monomer and induces DNA bending (9).

**Fig 6:**
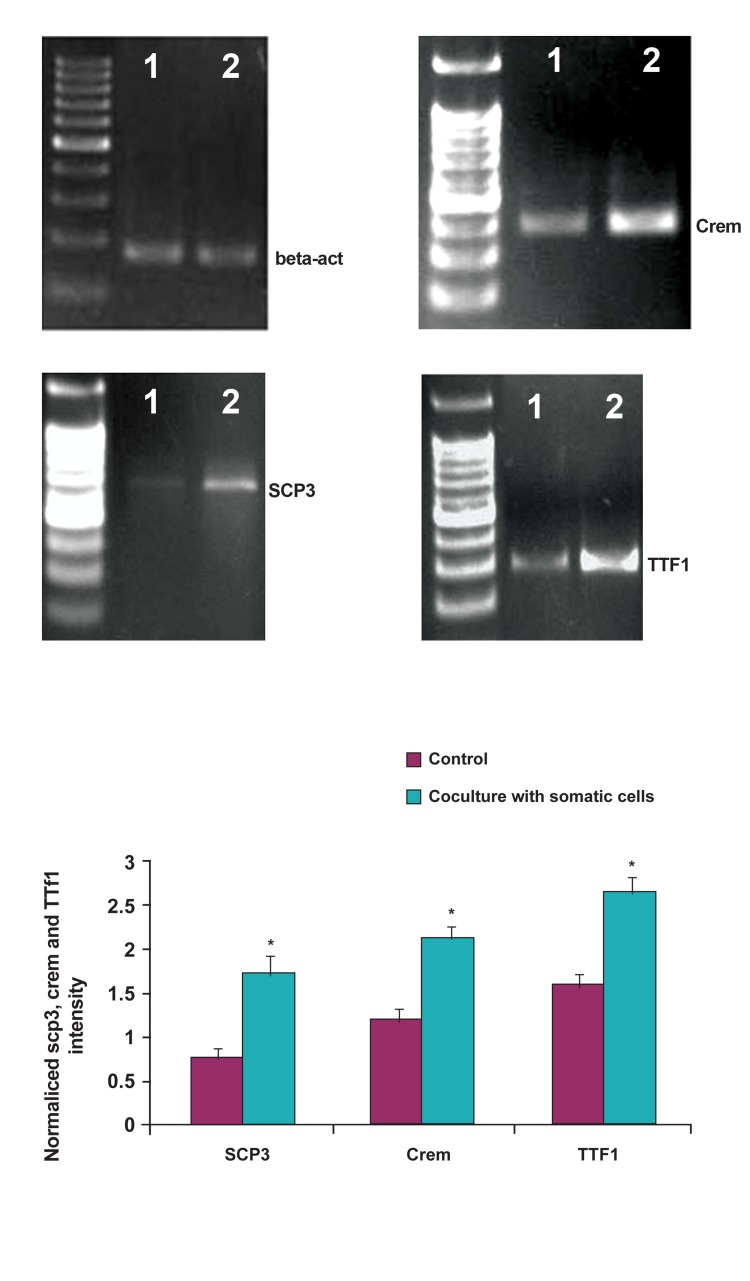
Expression analysis of meiotic and post meiotic marker genes Crem, TTf1 and SCP3.1, SSC culture without supporter cells 2, SSC culture with supporter cells. β-actin was used as an internal control. Densitometric analysis is shown in the histogram.

The expression levels of SCP3 showed marked increase in the colonies supported with somatic testicular cells in comparison with the colonies without these supporting cells. Two other post meiotic genes, Crem and TTF1, demonstrated significant increase in this group as well (Fig 6, p<0.05).

**Fig 7:**
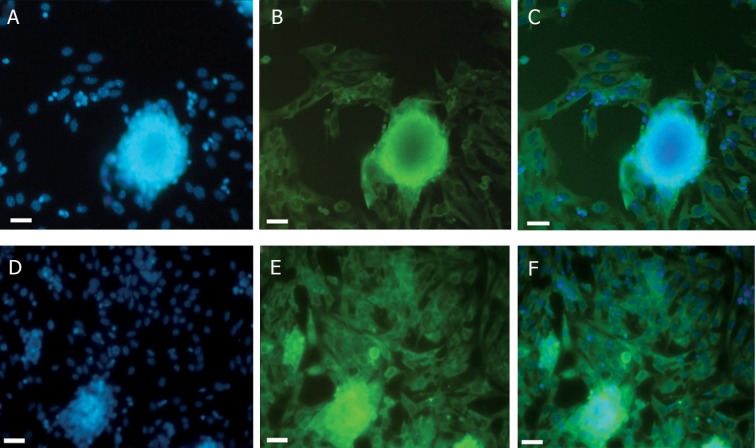
Representative immunofluorescence images (A) staining nucleus with DAPI (blue) and (B)showing SCP3 positive cells (green) (C) merge in control group (D, E, F) in co-culture group.

In order to investigate meiotic promotion in SSCs, antibodies against SCP3 were utilized. We observed that 6% of SSCs in the first group undergo meiosis 7 days after plating. In the second group in which SSCs were co-cultured with somatic testicular cells, the percentage of SCP3 positive cells increased dramatically. 13% of SSCs started meiosis in this group (Fig 7).

## Discussion

Spermatogenesis is characterized by mitotic (spermatogonia), meiotic (spermatocytes) and differentiative haploid (spermatids) phases and occurs in the seminiferous tubules of the testis. This highly complex process is under the control of endocrine system and also several autocrine and paracrine signals produced by surrounding microenvironment of SSCs (7). A complete spermatogenesis process starting from SSCs to spermatozoa differentiation has not been demonstrated in a culture system so far. The inability of SSC isolation and *in vitro* differentiation has caused a huge limitation in mature spermatozoa generation in a culture system (1).

Regarding the significant difference between juvenile and adult mice in SSCs population (10, 11), immature mouse testis has been utilized in this approach. The proportion of SSCs is up to 100-fold higher compared with adult testis (10). Some evidences hinted better spermatogonial viability (12) and differential potential in immature mice (13).

Owing to the small number of SSCs and lack of specific cell-surface markers, isolation of purified population of SSCs is extremely difficult (4). There are several approaches to isolate spermatogonia from testicular tissue (14-16). Previous studies confirmed that MACS system is the most suitable technique which causes minimal stress to the SSCs during isolation (17, 18). A specific cell surface marker which is expressed exclusively on undifferentiated SSCs lead to successful MACS isolation (19). Our flow cytometric and immunocytochemisteric analysis showed that Gfrɑ-1 is expressed exclusively in single spermatogonia and MACS can isolate a purified population of Gfrɑ-1 positive cells. Previously, Gfrɑ-1 had been introduced as an excellent marker for SSC isolation. It is expressed before starting the initial differentiation and expansion into pairs and chains (4). Our RT- PCR results showed higher expression of Gfrɑ-1 and OCT-4 as premeiotic specific markers after the isolation. This is in agreement with other studies which have demonstrated the double expression of Oct-3/4 and Gfrɑ-1 in type A spermatogonia (18, 20).

Previous studies suggested that male germ cells in a 3D culture system can be developed to the level of spermatids (4, 5). Recently, the generation of morphologically normal spermatozoa in SACS from mouse SSCs has been demonstrated (7). Detection of meiotic and post meiotic markers revealed that differentiation of SSCs in SACS prevents meiosis suppression which normally occurs under *in vitro* condition (7). A 3D culture approach was first introduced to characterize clonal expansion of bone marrow cells and to identify factors involved in their proliferation and differentiation (21, 22). Applied to SSCs, it has been suggested that 3D culture system can provide an appropriate microenvironment for clonal expansion of germ cells (5, 23).

Embedding SSCs in a 3D culture system in combination with somatic testicular cells provides a structure that mimics the complex structure found in living testes. Reaggregation of somatic testicular cells and SSCs in a collagen gel matrix might re-establish the proper contact of the cells and stimulate germ cell differentiation in the culture system. In addition, the similarity of collagen gel and extra cellular matrix (ECM) can provide an appropriate access to the structural proteins and biological molecules for the differentiating cells (5). Collagen gel matrix in a 3D culture system also can retain growth factors which are secreted by Sertoli cells and Sertoli cell morphology in a 3D culture system is closely similar to that of the seminiferous tubule (5, 7).

In this study, we focused on the possible role of testicular somatic cells in meiotic Gfrɑ-1 positive cells in a 3D culture of collagen gel. Before culturing and after the second digestion, we confirmed the presence of Sertoli cells and peritubular cells as testicular somatic cells in the cell suspension using ABP and α-Sm markers. Lower expression of these somatic markers after MACS isolation demonstrated again the efficacy of our isolation technique. This result is also in agreement with previous observations showing that a two-step digestion approach can provide somatic cells for 3D culture system most effectively (4, 7). Our data showed that the presence of these testicular cells in a culture system increase the mRNA levels of SCP3, Crem and TTF1 which are all involved in meiotic and post meiotic progression. It had been suggested that TTF1 plays important roles in chromatin condensation during spermatogenesis (9).

SSC differentiation is a coordinated process and therefore a two-dimensional culture system could not mimic the complexity of seminiferous epithelium which provides several components to the developing SSC differentiation during spermatogenesis (1). The complex niches in the male organ in which SSCs are located is supported by different types of somatic testicular cells. According to miscellaneous evidences, somatic cells have a critical role in stimulating spermatogenesis progression during long-term culture. Complex cell to cell interactions (germ cell-germ cell, germ cell-Sertoli cell, Peritubular-Sertoli cell) regulate spermatogenesis. Sertoli cells secrete many enzymes, transport proteins, adhesion molecules and regulatory factors which are immensely important for SSC differentiation. Indeed, Sertoli cells are nursing developing germinal cells and they are the only cells which directly interact with spermatogenic cells (24-26). Co-culture of Sertoli cells and Peritubular cells increases Sertoli cell survival dramatically and stimulates secretion of Sertoli cell products, such as transferrin, androgen-binding protein and lactate which are required for survival of germ cells (27).

In the present study we detected larger colonies in the presence of testicular cells. It seems that antiapoptotic factors produced by Sertoli cells have an important impact on SSC survival and colony formation in a culture system. Stem cell factor (SCF) produced by Sertoli cells, is a kind of paracrine growth factor and inhibits apoptotic events during spermatogenesis (28, 29). Regarding the antiapoptotic effects of SCF, it can be speculated that testicular cells have a positive effect on extensive colony formation.

We also revealed that somatic testicular cells exert a pro-differentiative effect in SSCs by increasing the percentage of meiotic nuclei in the cell culture and the mRNA levels of several genes which are involved in meiotic progression. Our findings were in agreement with previous observations which showed spermatogonial cell culture supported by testicular somatic cells improve propagation and differentiation of germ line cells (4, 7).

## Conclusion

If optimal culture conditions exist, meiosis can be initiated and completed in a culture system. In this study we presented data that collagen gel culture system supported by Sertoli and peritubular cells as somatic testicular cells provides an appropriate environment for the development of differentiating germ cells from pre meiotic into meiotic and post meiotic stages. Our preliminary findings indicate that 3D coculture system may induce spermatogenesis and optimize *in vitro* culture conditions.
